# Reverse effect of home-use binaural beats brain stimulation

**DOI:** 10.1038/s41598-023-38313-4

**Published:** 2023-07-08

**Authors:** Michal Klichowski, Andrzej Wicher, Agnieszka Kruszwicka, Roman Golebiewski

**Affiliations:** 1 Cognitive Neuroscience Center, Adam Mickiewicz University, Poznan, Poland; 2 Learning Laboratory, Faculty of Educational Studies, Adam Mickiewicz University, Poznan, Poland; 3 Department of Acoustics, Faculty of Physics, Adam Mickiewicz University, Poznan, Poland

**Keywords:** Human behaviour, Cognitive neuroscience, Cognitive control, Intelligence, Problem solving

## Abstract

Binaural beats brain stimulation is a popular strategy for supporting home-use cognitive tasks. However, such home-use brain stimulation may be neutral to cognitive processes, and any intellectual improvement may be only a placebo effect. Thus, without belief in it, it may bring no benefits. Here we test 1000 individuals at their homes as they perform a two-part fluid intelligence test. Some took the second part listening to binaural beats, while others took it in silence or listening to other sounds. The binaural beats group was divided into three subgroups. The first one was informed that they would listen to sounds that improve the brain's work, the second that neutral sounds, and the third that some sounds the nature of which was not defined. We found that listening to binaural beats was not neutral, as it dramatically deteriorated the score irrespective of the condition. Silence or other sounds had no effect. Thus, home-use binaural beats brain stimulation brings reverse effects to those assumed: instead of supporting the effectiveness of cognitive activities, it may weaken them.

## Introduction

Noninvasive brain stimulation (NIBS) techniques are currently very popular, both as tools for studying brain functions and strategies for supporting its functioning^1^. They include big and expensive devices that are only used in scientific or therapeutic laboratories, for example the equipment for transcranial magnetic stimulation (TMS), as well as small and relatively inexpensive ones that can be used at home. An example of the latter are transcranial direct current stimulation (tDCS) devices^2^. Business dedicated to all sorts of home-use neuromodulators is thriving, a sign of which is the growing number of online brain shops that offer devices for home-use improvement of brain functioning^3^. tDCS devices aimed at home use in order to—according to their producers—improve focus, attention, memory, and productivity can also be found in online markets at a very low price. The movement of neurohackers or brain hackers^4^ has also been quickly developing recently; on the Internet, they show how to build tools for brain stimulation and how to use such forms of do-it-yourself (DIY) brain stimulation^5,6^. An incredibly widespread brain hacking tool is auditory brain stimulation, in particular binaural beats brain stimulation^7^. In this case, a simple computer and a basic program for sound processing are sufficient to build a modulator. What is more, an audio file for brain stimulation can be placed on the Internet, e.g., on audio/video sharing platforms^8^. In order to stimulate, a headset connected to a smartphone or computer will suffice. On one of the most popular online video-sharing websites alone, there is therefore a huge number of binaural beats recordings labelled as “genius brain frequency” or “reach super focus”. On the web, there are numerous guidelines on how to use them at home in order to support the process of learning (e.g., when preparing for an exam), solve problems (e.g., when working from home) or carry out other cognitive tasks. They suggest that these activities should be carried out every day while listening to accurately selected binaural beats, which is why the recordings available on the Internet are very long (from several dozen minutes to a dozen hours). Such conditions are completely different from those known from scientific research into binaural beats when exposure to binaural beats is very short (from several seconds to several minutes) and accidental (most often linked to one visit to the laboratory)^9–11^. There is thus a question of whether home-use binaural beats brain stimulation is safe, as well as whether it is effective at all^12^.

The phenomenon of binaural beats occurs when tones with slightly different frequencies are presented separately to the right and left ear. The phase-locking effect is mainly responsible for the mechanism of binaural beats. This phenomenon involves the neuron generating action potentials at well-defined phases of a periodic acoustic signal^13,14^. Propagating in the nerve fibres of the auditory nerve, the action potentials encode temporal fine structure (TFS) information of the acoustic signal^15,16^. Decoding the information contained in the TFS considerably supports the pitch perception process. It can therefore be assumed that, due to the crossing of the neural pathways of the left and right ear, the action potentials in which the TFS information of the tones is contained interact and, consequently, the impression of binaural beats is created. It is hypothesized that the neural interaction effects produced in this way can influence/modify brainwave parameters in such a way that a beat frequency (the mismatch between the tones) synchronizes cortical oscillations and, consequently, can act supportively with respect to a given activity. In order to do so, a beat frequency is selected so that it is close to (or equal to) the target brain wave frequencies. Thus, to support cognitive processes associated with beta waves, for example, binaural beats with a beat frequency in the range of 13 to 30 Hz are used (e.g., 240 Hz to the right ear and 255 Hz to the left ear in the case of 15 Hz binaural beats brain stimulation). To modulate brain activity close to meditation, on the other hand, the theta range is used (from 4 to 8 Hz, e.g., 240 Hz to the right ear and 245 Hz to the left ear in the case of 5 Hz binaural beats brain stimulation). There are also binaural beats dedicated to other frequencies, such as delta (a beat frequency in the range between 2 and 4 Hz), alpha (8–13 Hz) and gamma (30 Hz and above)^10,17,18^. Nevertheless, the research results do not conclusively resolve whether binaural beats have a function that truly modulates brain activity, much less whether they have the potential to enhance cognitive processes^19–21^.

However, it has been suggested that frequent, prolonged and high-volume use of binaural beats may be detrimental to hearing^22^ and mental health^23^. Furthermore, the systematic use of binaural beats in order to improve cognitive processes (or relaxation, for example with the use of the so-called marijuana binaural beats^24^) bears the risk of stimulations becoming a form of digital drugs^3,22^ or—as is already the case with tDCS—brain doping^3,25^. Additionally, it has also been suggested that the improvement of complex cognitive processes, such as remembering, learning or problem solving, that is experienced while listening to binaural beats may not be a result of neuromodulation, but positive expectations^26^. It is highly probable because placebo effects are quite frequently observed in neurostimulation trials^3,27^. It refers to both clinical studies with large TMS-type equipment and patients (for example suffering from migraines)^28^, as well as laboratory experiments with small tDCS devices and healthy volunteers, related to e.g. improving cognitive performance or working memory (placebo-like effects have also been found in clinical tDCS trials where, for example, individuals with overweight and obesity participated^29^)^30–33^. There is no research, however, into the placebo effect when it comes to home-use NIBS aimed at facilitating everyday cognitive activities, for example by listening to binaural beats on one’s headphones while learning^34,35^. Yet, many studies have indicated that the positive effects of various forms of brain training commonly used at home^36^, for example brain games, are linked to the fact that those who use them expect to improve and believe in brain training effectiveness rather than to any mechanisms inherent in such training *per se*^37–41^. It seems that the same applies to home-use binaural beats brain stimulation. Therefore, in the present work, we propose the following hypothesis: the impact of home-use binaural beats brain stimulation remains neutral when one’s belief in it is not induced.

**Figure 1:**
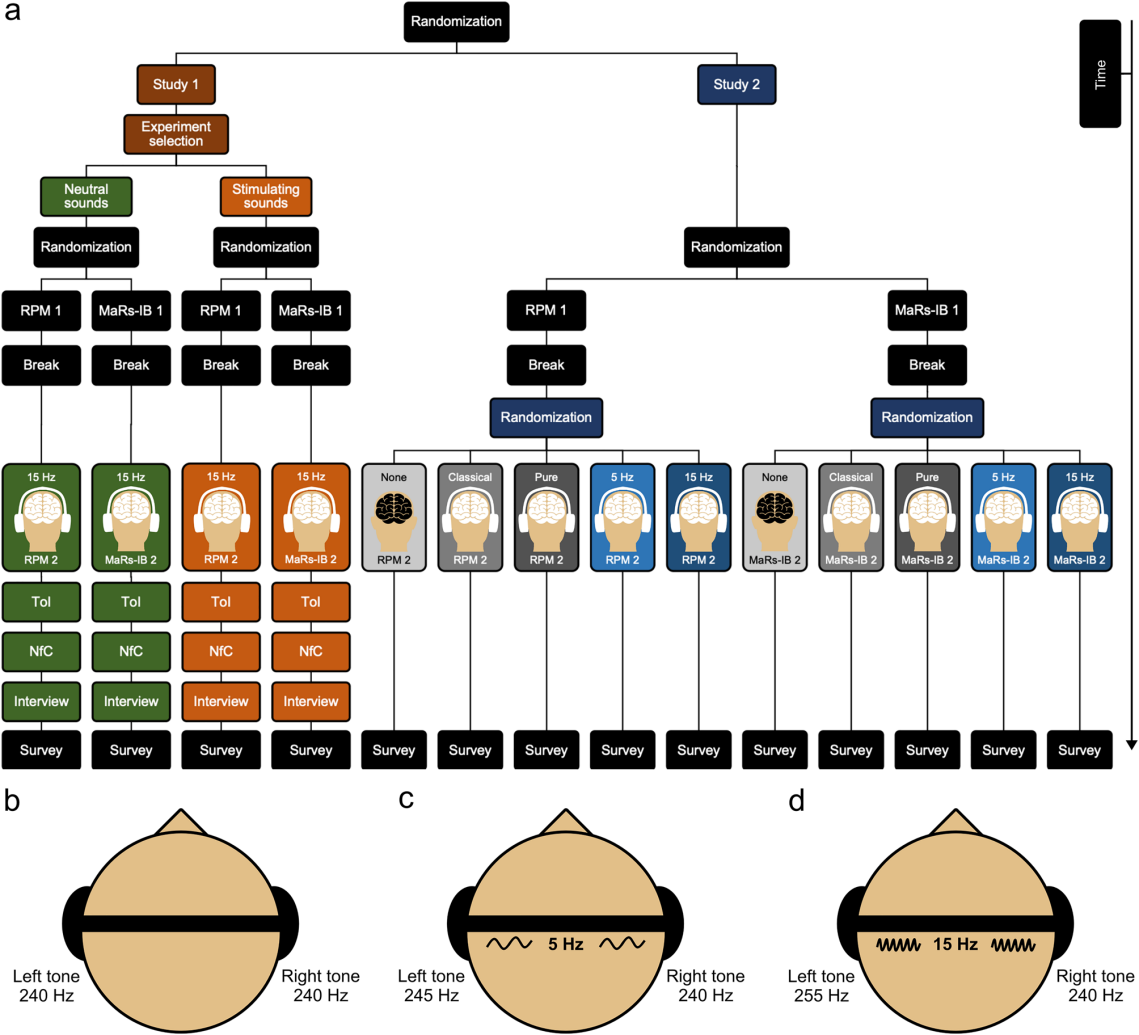
Schematic overview of the experimental workflows. (**a**) Study 1 and 2 design. Volunteers were randomly assigned to Study 1 or Study 2. They were instructed to sit down at their homes in a place where they most often carry out cognitive tasks on a phone or other device. Then a test to perform was randomly selected. It could have been the Raven's Progressive Matrices test (RPM) or the Matrix Reasoning Item Bank test (MaRs-IB). In Study 1, participants could choose whether to listen to neutral or brain-stimulating sounds during the experiment. There was a short break after the first part of the test, and the second part began. In Study 1, participants took this part while listening to 15 Hz binaural beats, irrespective of their chosen sounds. In Study 2, participants received information that the second part of the test would be completed in silence or while listening to "some sounds". The program randomly chose classical music, pure tone, 5 Hz binaural beats, or 15 Hz binaural beats. Finally, each participant in both studies filled out a demographic survey. Participants in Study 1 also completed two scales: the Theories of Intelligence scale (ToI) and the Need for Cognition scale (NfC). They, moreover, answered the question about the reason for participating in a given type of experiment. This procedure allowed us to examine home-use binaural beats brain stimulation effects when presented as sounds that improve the brain's work, as neutral sounds, or as sounds whose nature is not defined. We could also confront these effects with those of silence, other acoustic stimuli, and binaural beats not used for cognitive enhancement. Ultimately, we tested some potential moderators for the effects of such neuromodulation, e.g., beliefs about cognition and intelligence. (**b**) Pure tone used in Study 1. (**c**) 5 Hz binaural beats brain stimulations of Study 2. (**d**) 15 Hz binaural beats brain stimulations used in both studies.

In order to check this hypothesis, we carried out two experiments (Fig. 1a) in which altogether 1000 adult individuals of different ages and characteristics participated. In the first one, we partially repeated the procedure of a classic study that demonstrated placebo effects in brain training^41^, and we changed the experimental factor from brain training to home-use binaural beats brain stimulation. Volunteers would sit down at their homes in a place and time when they most often carry out various cognitive tasks on a computer or phone. Then, they would run a digital questionnaire on their preferred device and choose which study they wanted to participate in. They could choose a “basic study” whose description suggested that in its second part they would listen to neutral sounds on their headphones or a “study with brain stimulation” whose description suggested that in its second part they would listen to sounds which, as indicated by numerous research, improve brain activity and, as a consequence, increase the effectiveness of carrying out cognitive tasks. After making their choice of the study, the program randomly selected which of the two fluid intelligence tests (Raven’s Progressive Matrices test which takes about an hour or Matrix Reasoning Item Bank test which takes about 40 min) the participant would take. Both tests consist in identifying relationships between abstract shapes and are built in a way that makes it possible to divide them into two parts of equal levels of difficulty. As a result, when taking them under identical conditions, a participant should have the same score on each part (in some studies, a slight improvement in the second part was observed as a practice effect, but it was a trend rather than a significant difference)^41–43^. On the other hand, when using some effective cognitive enhancement interventions, the score on the second part should be considerably better (or improvement in the intervention group should be significantly greater than in the control one)^41,44^. After taking the first part of the test, the participants were supposed to take a short break after which they would put the headphones on and complete a task checking if they were put on correctly, as well as set the volume comfortably. Then, they would take the second part of the test listening to 15 Hz binaural beats (binaural beats most often used for improving focus or effectiveness of learning^17^) irrespective of the type of study they chose. After finishing the task, like in the replicated study, they would complete two additional tests related to beliefs about cognition and intelligence, because these metrics are potential moderators for the effects of neuromodulation. Finally, apart from a demographic survey, each participant would justify in writing why they chose a given type of study. In the other study, in order to follow methodological standards^45^ and also have other/control acoustic interventions apart from the binaural beats intervention, we used stimuli from a well-known experiment on the effectiveness of binaural beats brain stimulation^18^, implementing them in the Study 1 procedure. In Study 2, however, the participant did not choose the type of study; instead, the program drew the test right after running the electronic questionnaire. The participant would only receive information that the second part of the test would be completed while listening to “some sounds”. After the first part of the test was completed, the program would draw whether it would be classical music, pure tone (Fig. 1b), 5 Hz binaural beats (binaural beats most often used for relaxation^17^; Fig. 1c), 15 Hz binaural beats (Fig. 1d) or silence (information would then be displayed that headphones are not necessary after all). Finally, each participant would fill out a demographic survey (see Methods for additional details). We assumed that in Study 1 we would not observe increased test scores in the group in which 15 Hz binaural beats were presented as neutral sounds, with possible improvement in the second group (given our procedure, where the placebo effect is not strongly induced, but binaural beats is just presented as stimulating sounds, there may be no effect here either), irrespective of the test taken. In Study 2, we did not expect improvement in the score in any condition. Such results would confirm the hypothesis that home-use binaural beats brain stimulation is neutral to cognitive processes. However, our results do not confirm these assumptions and suggest that binaural beats may negatively affect home-use cognitive activity.

## Results

### Effects of home-use binaural beats brain stimulation

Four hundred individuals were tested in Study 1. The criterion of being taken into account in analyses, i.e., accuracy in the first (baseline) part of the test on the level of minimum 22.22% (this value reflects completing 4 out of 18 tasks correctly; a lower score among healthy adults reflects misunderstanding the instructions or choosing random answers^46^) was met by 369 individuals. Although more individuals decided to take part in the study in the stimulating sounds version (57.45%), the groups were balanced with respect to all basic characteristics, such as age, gender, place of residence and everyday activity (see Methods for more details). Given our hypothesis that an improved test score in Study 1 would not be observed in the group in which binaural beats were presented as neutral sounds, with possible improvement in the second one (in which binaural beats were presented as stimulating sounds), we initially tested the main effect of time. 2 × 2 × 2 ANOVA with the time (baseline, intervention) as within-subjects factors, and the group (Neutral Sounds, Stimulating Sounds) and test (Raven’s Progressive Matrices test, Matrix Reasoning Item Bank test) as between-subjects factors revealed a significant main effect of the time and no significant time × group, time × test, or time × group × test interactions (see Supplementary Table 1 for more details). To our surprise, the effect of the time consisted of a significant deterioration of the score by nearly 6% (the mean difference = 5.86, *SE* = 0.69,* t* = 8.498, *df* = 365, *Tukey's p* < 0.00001).

**Figure 2:**
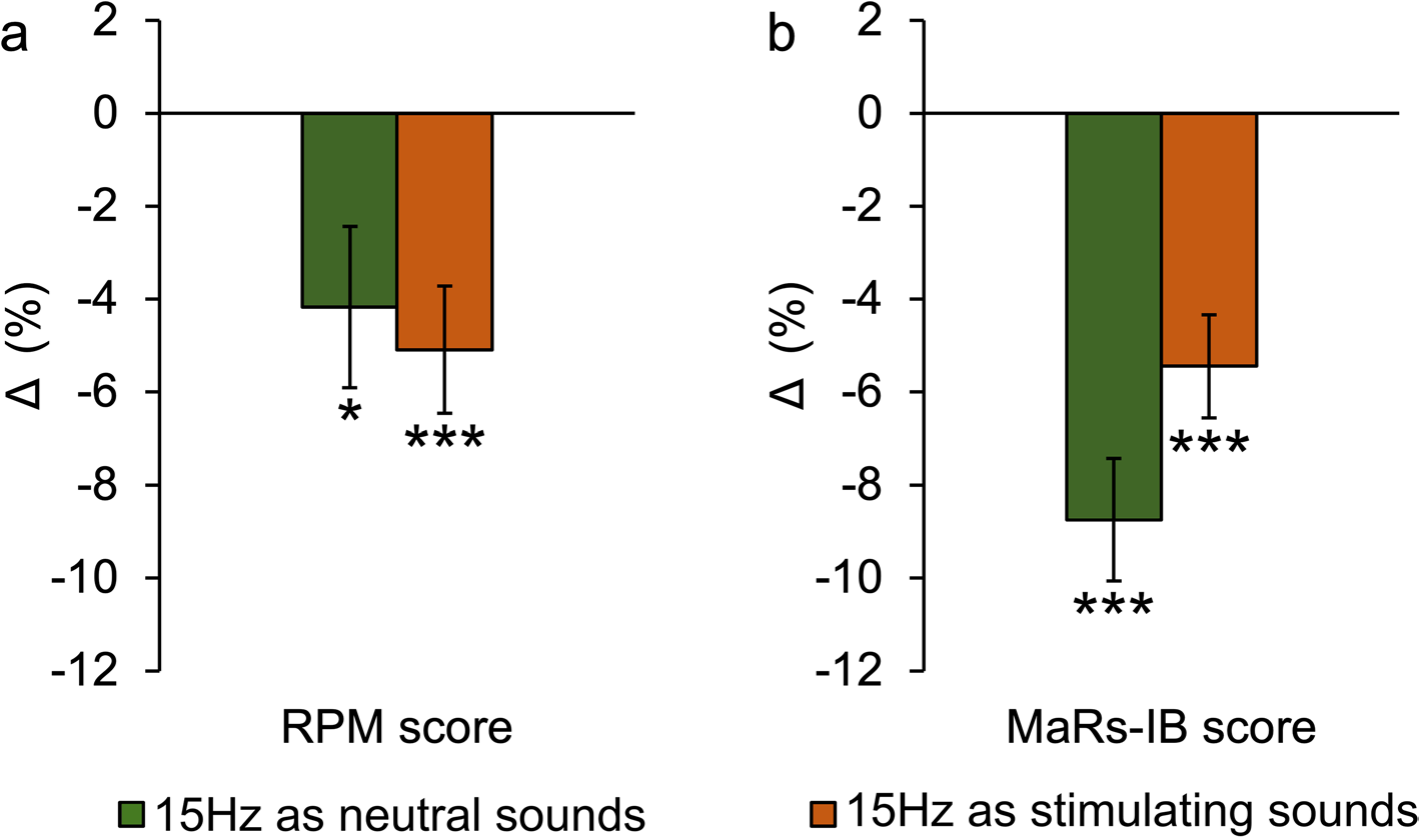
Home-use binaural beats (15 Hz) brain stimulation effects on the score of fluid intelligence tests under two conditions (15 Hz binaural beats presented as neutral sounds and 15 Hz binaural beats presented as stimulating sounds). (**a**) Paired samples *t*-tests show the significant influence of the binaural beats intervention on Raven’s Progressive Matrices score regardless of the condition (*n* = 175). (**b**) Paired samples *t*-tests show the significant influence of the binaural beats intervention on Matrix Reasoning Item Bank score regardless of the condition (*n* = 194). Changes/deltas (Δ) were calculated by the formula: intervention scores − baseline scores (0 = the no-stimulation baseline scores). Asterisks indicate significant differences with *p*-values of *p < 0.05, ***p < 0.001. Error bars depict standard errors of the means. Source data are provided as a Source Data file deposited in the Open Science Framework database (https://osf.io/kp48h).

In the next step, we calculated the value of the change/delta (Δ) by subtracting the percentage score of the second part of the test from the percentage score of the first part of the test. Thanks to that, the value obtained shows to what extent (by how many per cent) the intervention influenced the score. A positive value shows an improvement in the score, while a negative one indicates its deterioration. In order to determine whether the change on the group level is significant, we compared the mean group score from the baseline part with the mean score from the part with the intervention using a two-tailed paired samples *t*-test. As depicted in Table 1, the intervention of 15 Hz home-use binaural beats brain stimulation considerably lowered each group’s scores. In the case of the longer Raven’s Progressive Matrices test, the Neutral Sounds group score deteriorated by over 4%, and the Stimulating Sounds one by slightly over 5% (Fig. 2a). On the other hand, in the shorter Matrix Reasoning Item Bank test the deterioration was of nearly 9% and considerably over 5%, respectively (Fig. 2b). However, as revealed by a 2 × 2 ANOVA with the group (Neutral Sounds, Stimulating Sounds) and test (Raven’s Progressive Matrices test, Matrix Reasoning Item Bank test) as factors, the difference between the tests in how much the scores deteriorated was insignificant (Table 2). What is more, we found no significant main effect of the group. This means that listening to 15 Hz binaural beats while taking the test deteriorated its score as compared to the baseline test, even if their use is accompanied by a suggestion that the sounds are neutral or help carry out cognitive tasks. The outcomes, therefore, suggest that home-use binaural beats brain stimulation leads to reverse effects, i.e., instead of helping one carry out a cognitive task, it hinders it in some sense.

**Table 1:** Home-use binaural beats brain stimulation worsens the score of fluid intelligence tests under both conditions.

Test	Group	Score (%)		Δ (*SE*)	*t*	*df*	*p*	*d*	1 − *β*
		Baseline (*SE*)	Intervention (*SE*)						
RPM	15 Hz NS (*n* = 68)	47.96 (2.05)	43.79 (2.41)	− 4.17 (1.74)	− 2.401	67	0.019*	− 0.291	0.657
	15 Hz SS (*n* = 107)	53.22 (1.94)	48.13 (2.16)	− 5.09 (1.37)	− 3.724	106	0.00032***	− 0.360	0.958
MaRs-IB	15 Hz NS (*n* = 89)	54.62 (2.46)	45.88 (2.77)	− 8.74 (1.32)	− 6.623	88	0.00001***	− 0.702	1.000
	15 Hz SS (*n* = 105)	62.49 (2.58)	57.04 (2.84)	− 5.45 (1.11)	− 4.910	104	0.00001***	− 0.479	0.998

Paired samples *t*-test indicating the influence of the binaural beats (15 Hz) intervention on the score of fluid intelligence tests. *RPM* Raven’s Progressive Matrices, *MaRs-IB* Matrix Reasoning Item Bank, *NS* neutral sounds, *SS* stimulating sounds, *Δ* change/delta calculated by the formula: intervention scores − baseline scores. Standard errors are in parentheses. Asterisks indicate significant differences with *p*-values of *p < 0.05, ***p < 0.001.

**Table 2:** Home-use binaural beats brain stimulation worsens the scores of both fluid intelligence tests to the same extent regardless of the condition.

	Sum of squares	Mean square	*F*	*df*	*p*	*η*^*2*^*p*
Group	125	125	0.737	1	0.391	0.002
Test	543	543	3.198	1	0.075	0.009
Group × test	396	396	2.330	1	0.128	0.006
Residuals	61,998	170		365		

2 × 2 ANOVA indicating the differences in the influence of the binaural beats (15 Hz) intervention on the fluid intelligence test score by condition (group) and test type (*n* = 369).

### Home-use binaural beats brain stimulation and beliefs and demographic characteristics

The scale of the score deterioration observed in Study 1 as far as the home-use fluid intelligence test carried out under binaural beats brain stimulation is concerned was not moderated by beliefs about cognition and intelligence. The Pearson correlation analysis showed that the calculated values of the change/delta (Δ) were not correlated with the values of the Theories of Intelligence scale (*r* = 0.032, *p* = 0.536) or the Need for Cognition scale (*r* = 0.050, *p* = 0.334) (see Supplementary Figs. 1 and 2). Furthermore, we did not find a correlation with the age of the participants (*r* = − 0.028, *p* = 0.587) (see Supplementary Fig. 3). Other demographic characteristics did not diversify the size of the change, either. Subsequent analyses of variance yielded no effect of the gender (*F*_2,366_ = 0.585, *p* = 0.558), residence (*F*_2,366_ = 0.418, *p* = 0.659), status / everyday activity (*F*_3,365_ = 0.968, *p* = 0.408) or education (*F*_3,365_ = 2.120, *p* = 0.097). The device chosen by participants to carry out the test (computer, smartphone, tablet or TV set) did not diversify the change, either (*F*_3,365_ = 0.354, *p* = 0.786) (see Supplementary Table 2 for more details). Additionally, a two-tailed independent samples *t*-test revealed no significant difference in the Theories of Intelligence scale and Need for Cognition scale when comparing the Neutral Sounds and Stimulating Sounds groups (*t*_367_ = − 0.650, *p* = 0.516 and *t*_367_ = − 1.238, *p* = 0.217, respectively; see Supplementary Table 3 for additional details). This indicates that the choice to participate in a study on brain stimulation was not moderated by the need for cognition and beliefs about intelligence.

The analysis of justifications for the study choice shows that slightly more than half of the participants selected neutral sounds background justified their choice with their curiosity about its course, and every tenth with a simple will to carry out an easier task. Some participants (~ 10%) also indicated that they were afraid of stimulation or (~ 5%) made a random choice (~ 10% gave an answer that was difficult to classify, and just over 5% gave no answer). Nearly three-quarters of participants selected auditory brain stimulation due to their cognitive curiosity about brain stimulation. Many participants from this group also underlined that they liked cognitive challenges, and the study with stimulation looked like one. Two participants added that they felt stimulation disturbed them in the task. Some participants (about 5%) indicated that their choice was random, and less than 5% wrote something that should be indicated as no answer. About one-fifth of participants gave justifications that are difficult to classify (see Supplementary Table 4 for justification examples). These qualitative results indicate that most participants chose the study type consciously, following their own needs or convictions. When the study with brain stimulation was selected, it was most often linked to the will to experience stimulation or curiosity caused by the topic of brain stimulation. Thus, it can be assumed (although the limitations of this qualitative analysis must be taken into account) that most of the Stimulating Sounds experiment participants did not have negative attitudes towards stimulation.

### Home-use binaural beats brain stimulation and other acoustic stimulations

Six hundred individuals were tested in Study 2. The criterion of being taken into account in analyses (the same as in Study 1, i.e., accuracy in the baseline part = minimum 22.22%) was met by 551 individuals. The groups did not differ with respect to all basic characteristics, such as age, gender, place of residence and everyday activity (see ﻿Methods for additional details). Exactly like in Study 1, we initially tested the main effect of time. 2 × 5 × 2 ANOVA with the time (baseline, intervention) as within-subjects factors, and the group (None, Classical, Pure, 5 Hz, 15 Hz) and test (Raven’s Progressive Matrices test, Matrix Reasoning Item Bank test) as between-subjects factors revealed a significant main effect of the time and significant time × group interaction. There were no other significant interactions (see the top panel of Supplementary Table 5 for more details). As in Study 1, the effect of the time consisted of a significant deterioration of the score, but here only by nearly 3% (the mean difference = 2.69, *SE* = 0.57,* t* = 4.69, *df* = 541, *Tukey's p* < 0.00001). However, estimated-marginal-means-based post-hoc comparisons (bottom panel of Supplementary Table 5) showed that this deterioration applies only to binaural beats brain stimulation groups. These results contradict our hypothesis that we would not observe a significant change in the score in any acoustic condition.

**Figure 3:**
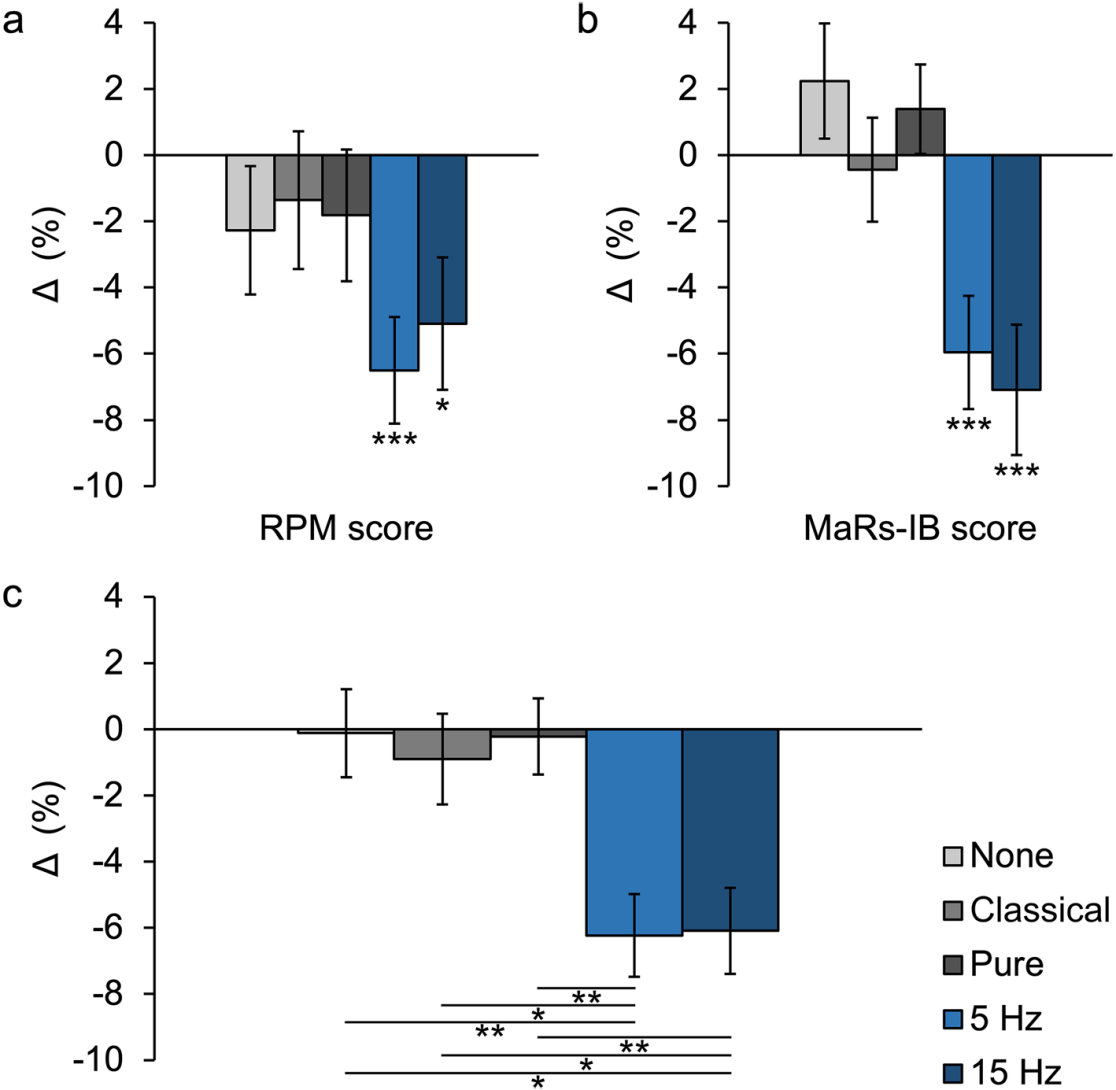
Home-use binaural beats brain stimulations vs. other acoustic stimulations effects on the score of fluid intelligence tests. (**a**) Paired samples *t*-tests show the significant influence of the binaural beats interventions on Raven’s Progressive Matrices score and no such effects for other acoustic stimulations (*n* = 268). (**b**) Paired samples *t*-tests show the significant influence of the binaural beats interventions on Matrix Reasoning Item Bank score and no such effects for other acoustic stimulations (*n* = 283). **c** Estimated-marginal-means-based post-hoc comparisons show that the binaural beats interventions worsen the scores of both fluid intelligence tests to the same extent and their influence is significantly worse than in the case of other acoustic stimulations (*n* = 551). Changes/deltas (Δ) were calculated by the formula: intervention scores − baseline scores (0 = the no-stimulation baseline scores). Asterisks indicate significant differences with *p*-values of *p < 0.05, **p < 0.01, ***p < 0.001. Error bars depict standard errors of the means. Source data are provided as a Source Data file deposited in the Open Science Framework database (https://osf.io/kp48h).

Using a two-tailed paired samples *t*-test (Table 3) we compared each group’s average score from the baseline part with the average score from the part with the intervention. Again, we found that an intervention of both 5 Hz and 15 Hz home-use binaural beats brain stimulation deteriorated the score significantly. In the case of the longer Raven’s Progressive Matrices test, the score in the 5 Hz group deteriorated by over 6%, and in the 15 Hz one by slightly over 5% (Fig. 3a). On the other hand, in the shorter Matrix Reasoning Item Bank test, the deterioration was almost 6% for 5 Hz and slightly over 7% for 15 Hz (Fig. 3b). In the case of other conditions, the differences between the baseline and intervention were insignificant. Further, we calculated the value of the change/delta (Δ) by subtracting the score of the second part of the test from the result of the first part of the test and ran 5 × 2 ANOVA with the group (no sounds, classical music, pure tone, 5 Hz binaural beats, 15 Hz binaural beats) and test (Raven’s Progressive Matrices test, Matrix Reasoning Item Bank test) as factors. It revealed no significant main effect of the test and a significant main effect of the group (top panel of Table 4). Estimated-marginal-means-based post-hoc comparisons (bottom panel of Table 4) showed no significant differences in the score between two types of binaural beats brain stimulation and significant differences between these stimulations and other acoustic conditions, always consisting in worse scores for the binaural beats condition (Fig. 3c). This means that listening to both types of binaural beats (both the one that is supposed to be relaxing and the one that is supposed to stimulate cognitive processes) when taking the test lowered its score as compared to the baseline test, and listening to other sounds (or silence) had on influence. These scores again suggest that home-use binaural beats brain stimulation hinders the completion of cognitive tasks instead of fostering it. We thus observed reverse effects of home-use binaural beats brain stimulation again.

**Table 3:** Home-use binaural beats brain stimulations worsen the score of fluid intelligence tests while other acoustic stimulations do not affect them.

Test	Group	Score (%)		Δ (*SE*)	*t*	*df*	*p*	*d*	1 − *β*
		Baseline (*SE*)	Intervention (*SE*)						
RPM	None (*n* = 49)	56.58 (2.99)	54.31 (3.21)	− 2.27 (1.94)	− 1.171	48	0.247	− 0.167	0.209
	Classical (*n* = 45)	55.80 (3.29)	54.44 (3.58)	− 1.36 (2.08)	− 0.654	44	0.516	− 0.098	0.099
	Pure (*n* = 67)	54.31 (2.25)	52.49 (2.7)	− 1.82 (1.99)	− 0.919	66	0.362	− 0.112	0.147
	5 Hz (*n* = 59)	58.00 (2.52)	51.51 (2.71)	− 6.50 (1.61)	− 4.043	58	0.00016***	− 0.526	0.978
	15 Hz (*n* = 48)	63.08 (3.00)	57.99 (3.66)	− 5.09 (2.00)	− 2.549	47	0.014*	− 0.368	0.704
MaRs-IB	None (*n* = 52)	72.01 (3.30)	74.25 (3.36)	2.24 (1.74)	1.288	51	0.203	0.179	0.245
	Classical (*n* = 50)	64.67 (3.57)	64.22 (3.91)	− 0.44 (1.57)	− 0.283	49	0.778	− 0.040	0.059
	Pure (*n* = 68)	60.54 (3.13)	61.93 (3.65)	1.39 (1.35)	1.029	67	0.307	0.125	0.174
	5 Hz (*n* = 55)	58.99 (3.39)	53.03 (4.05)	− 5.96 (1.71)	− 3.483	54	0.00099***	− 0.470	0.928
	15 Hz (*n* = 58)	61.30 (3.26)	54.22 (4.22)	− 7.09 (1.97)	− 3.599	57	0.00067***	− 0.473	0.943

Paired samples *t*-test indicating the influence of the type of intervention on fluid intelligence tests scores. *RPM* Raven’s Progressive Matrices, *MaRs-IB* Matrix Reasoning Item Bank, *Δ* change/delta calculated by the formula: intervention scores − baseline scores. Standard errors are in parentheses. Asterisks indicate significant differences with *p*-values of *p < 0.05, ***p < 0.001.

**Table 4:** Home-use binaural beats brain stimulations worsen the scores of both fluid intelligence tests to the same extent and their influence is significantly worse than in the case of other acoustic stimulations.

	Sum of squares	Mean square	*F*	*df*	*p*	*η*^*2*^*p*	
Group	4492	1123	6.295	4	0.00006***	0.044	
Test	279	279	1.564	1	0.212	0.003	
Group × Test	683	171	0.957	4	0.430	0.007	
Residuals	96,517	178		541			

Post-hoc comparisons		Mean difference (*SE*)	*t*	*df*	Tukey's *p*	*d*	1 − *β*
15 Hz (*n* = 106)	None (*n* = 101)	− 6.08 (1.86)	− 3.265	541	0.010*	− 0.455	0.903
	Classical (*n* = 95)	− 5.19 (1.89)	− 2.742	541	0.049*	− 0.389	0.782
	Pure (*n* = 135)	− 5.87 (1.74)	− 3.380	541	0.007**	− 0.440	0.922
	5 Hz (*n* = 114)	0.14 (1.81)	0.076	541	1.000	0.010	0.051
5 Hz (*n* = 114)	None (*n* = 101)	− 6.22 (1.83)	− 3.404	541	0.006**	− 0.465	0.923
	Classical (*n* = 95)	− 5.33 (1.86)	− 2.868	541	0.035*	− 0.399	0.816
	Pure (*n* = 135)	− 6.01 (1.7)	− 3.537	541	0.004**	− 0.450	0.941
Classical (*n* = 95)	None (*n* = 101)	− 0.89 (1.91)	− 0.465	541	0.990	− 0.067	0.075
	Pure (*n* = 135)	− 0.68 (1.79)	− 0.382	541	0.995	− 0.051	0.067
None (*n* = 101)	Pure (*n* = 135)	0.21 (1.76)	0.117	541	1.000	0.015	0.052

5 × 2 ANOVA indicating the main differences in the influence of the type of intervention on the fluid intelligence test score by condition (group) and test type. Asterisks indicate significant differences with *p*-values of ***p < 0.001 (*n* = 551). Estimated-marginal-means-based post-hoc comparisons indicating the differences in the influence of the type of intervention on the score of fluid intelligence tests. Standard errors are in parentheses. Asterisks indicate significant differences with *p*-values of *p < 0.05, **p < 0.01.

## Discussion

Despite a growing body of work on NIBS and increasing interest in using some forms of such stimulation to support healthy individuals’ cognitive processes, there is still a lack of research into applying them in this way. It refers especially to home-use NIBS, for example in the form of listening to binaural beats while learning or completing other cognitive activities at home which is incredibly popular. In our study, we examined, for the first time, the effectiveness of home-use binaural beats brain stimulation. We assumed that this efficacy is a function of users’ convictions and that the positive influence of binaural beats on cognitive performance would not be observed in other than placebo-like conditions. Our results, however, show something completely different. Home-use binaural beats brain stimulation not only did not support cognitive processes, but it caused a reverse effect by worsening the scores of cognitive activities. Although further research into this phenomenon is required, our results challenge the conventional thinking about using NIBS at home and DIY brain stimulation and shed light on other matters of contention, such as the negative impact on cognitive processes. In other words, our project shows that home-use brain stimulation is not neutral to cognitive processes, which may bring placebo-like cognitive benefits, if any, and pose a threat to physical or mental health. It may also be detrimental to the effectiveness of cognitive processes themselves and bring results that are contrary to what is planned.

Thereby, our results are consistent with evidence that binaural beats brain stimulation does not have any special potential as far as enhancing cognitive functions is concerned^47^. Yet, they challenge the hypothesis of the no influence/neutral effect of home-use binaural beats brain stimulation on cognitive activities, as they show that it hindered the completion of the tasks (Fig. 2). The deterioration observed could not have been the nocebo effect (for example frequently observed in tDCS studies^3,48^), because it occurred in all conditions, i.e., also when the nature of sounds was not defined. It did not apply to a specific group of participants, either, as it was not moderated with any individual characteristics, such as age, gender, place of residence, education and everyday activity, or convictions about cognition and intelligence. This would suggest that the reverse effect of home-use binaural beats brain stimulation may be a universal phenomenon, i.e., apply to all groups of people. In our study, however, only adults took part; further research is required with younger participants, especially youths, as they form a large group of those using brain stimulation while learning at home. It would also be recommended to take into account other individual characteristics that may fulfil a moderating role, such as, for example, individual cognitive-control systems^49^. Having no such research, the universal nature of the reverse effect of home-use binaural beats brain stimulation is purely a hypothesis. What is more, creating conditions of an even larger ecological validity by replacing fluid intelligence tests with real-life tasks and adding conditions with other forms of NIBS, like e.g., tDCS, would provide an opportunity to tackle the issue of home-use brain stimulation even better.

Research that will explain the mechanism responsible for the reverse effect of home-use binaural beats brain stimulation is also in demand. In our study, it was present both in the case of stimulation based on 15 Hz binaural beats, whose premise is to modulate beta rhythms leading to enhanced cognitive functions, and 5 Hz, which is supposed to modulate theta rhythms, relax, and even put one to sleep^10,18,50^. The influence of both these stimulations was the same (Fig. 3c), which might suggest that prolonged listening to any binaural beats is to an extent bothersome and disturbing, but does not affect the work of the brain directly. On the other hand, listening to very similar sounds, i.e., pure tone, did not lower the score, which might rather suggest that the deterioration was due to some modulation of brain activity.

The binaural beats effect can be considered in terms of subjective evaluation of the auditory sensation (loudness fluctuations and moving of the auditory image in the participant's head). Also, it can be analyzed objectively by EEG recordings, e.g., measuring the amplitude envelope of EEG potentials whose modulation rate is equal to the binaural beats^51,52^. Such EEG evidence for the production of periodic changes in electrophysiological potentials with a frequency corresponding to binaural beats may be a starting point for the hypothesis regarding the mechanism of formation of the reverse effect of binaural beats brain stimulation. There seems to be an interaction between binaural beats waves and brain waves by analogy with the beating phenomenon. In our study, binaural beats of two frequencies, 5 Hz and 15 Hz, were formed in the participants. Since they were taking part in tests requiring active concentration and working memory involvement, it can be assumed that their brains were dominated by beta-type waves^53^, for which there is a relatively large range of frequency variability (from 13 to 30 Hz). Thus, at both 5 Hz and 15 Hz binaural beats, there may have been a phenomenon of "beating" of these waves with brain waves. As a result of this interaction, there may have been a "creation" of brain waves at low frequencies of 3–8 Hz, corresponding to theta waves. The brain generates theta waves during the conscious state of relaxation. Such a "forced" state of rest would certainly cause drowsiness^54^, affect concentration and impair performance on tasks requiring inductive reasoning and attention. This mechanism would explain the adverse effects of binaural beats on cognitive function found in this project. At the same time, the "beating effect" partly clarifies why many studies found no expected changes in brain wave recordings when the brain was stimulated via binaural beats^55,21^. Of course, there may also be no specific mechanism here. Fluid intelligence tests in some parts require conditional reasoning, which can be supported by gamma-band rather than beta-band stimulation^56^. Thus, although 15 Hz binaural beats can modulate beta oscillatory brain activity, they may also prevent temporary transitions to gamma frequencies, worsening test results and causing a reverse effect of stimulation.

Further research should thus monitor brain activity during home-use binaural beats brain stimulation, for example via EEG. This would naturally decrease the ecological validity of the study, so an EEG experiment could simply be carried out in a laboratory. It could subject the participants to a long (e.g., half-an-hour long) binaural beats brain stimulation, while simultaneously monitoring brain activity all the time or registering it every couple of minutes. It would have to take into consideration the suggestion that a few minutes of exposure are necessary in order to be able to observe the influence of binaural beats on the EEG record^9^. The measurement could perhaps take place before and after binaural beats brain stimulation.

To conclude, we did not confirm our hypothesis that home-use binaural beats brain stimulation is neutral to cognitive processes. Our results show that binaural beats brain stimulation can radically weaken the effectiveness of the home-use cognitive activity. Although further research is required, our project encourages a cautious approach to home-use NIBS. We recommend that home-use NIBS should be monitored as a possible threat to physical and mental health, as well as laboratory procedures of accidental stimulation transferred to home reality should be observed as to whether they bring the expected results. Even if recommended standards of use are followed and procedures that were successful in the laboratory extrapolated, we cannot be sure that NIBS does not affect the effectiveness of everyday cognitive activities.

## Methods

### Participants

One thousand volunteers took part in this study. Participants were recruited using electronic advertisements on the website and Facebook profile of Adam Mickiewicz University, Poznan. It was stated in the advertisement that the study can be participated in only by adult individuals who have no history of neurological disorders and no hearing problems, as well as normal or corrected-to-normal vision (each person starting the experiment had to declare meeting all of these criteria). Having finished the study, each participant was asked to invite one person of, for example, different gender or considerably different age to take part. This was meant to balance the group and make it possible to test more than just students. Eighty participants were excluded from further analyses because of demonstrating a failure to understand task instructions or choosing random answers based on accuracy errors, defined here as completing fewer than 4 out of 18 tasks in the first part of the test (i.e., baseline accuracy lower than 22.22%). The final sample thus consisted of 920 adult participants (496 women, 421 men and 3 non-binary gender; age range: 18–81, mean = 25.75, *SD* = 9.54). All were inhabitants of Poland, of which 29.78% lived in a city, 33.48% in a town and 36.74% in a village. They also differed with regard to their status / everyday activity. There were 65.11% students or pupils and 31.63% employees. Those unemployed or retired constituted 1.74%, and 1.52% were of other status. 29.24% of participants had higher education, 14.78% finished their education at the secondary level, and 6.63% at the primary or vocational level. The remaining individuals had not finished their education yet. Most individuals (72.83%) carried out the experimental tasks on a computer. A smartphone was used by 25.87%, a tablet by a scarce 1.09%, and a TV set by 0.22%.

The participants were randomly assigned to Study 1 or Study 2, with a limit of 400 participants in Study 1 and 600 in Study 2. We assumed that even with a considerable number of exclusions, we would be able to have a minimum of 40 individuals per condition, which is equivalent to the classic standard in intervention experiments^57^. Also, an a priori power analysis confirmed that a sample size of 40 in each group will be sufficient to demonstrate a medium effect size of 0.5 with Type I error (*α*) of 0.05 and Type II error (*β*) of 0.20 (1 – *β*/power = 80%). Finally, we had many more participants per condition—in the smallest group *n* = 45.

In Study 1 the participants chose the test type themselves. Both groups (called Neutral Sounds and Stimulating Sounds after the description of an acoustic stimulus) did not, however, differ with respect to their basic characteristics, such as age (*t*_367_ = 0.823, *p* = 0.411), gender (*χ*^*2*^ = 0.956, *df* = 2, *p* = 0.620), residence (*χ*^*2*^ = 1.910, *df* = 2, *p* = 0.386), status / everyday activity (*χ*^*2*^ = 3.574, *df* = 3, *p* = 0.311). The groups differed with respect to education (*χ*^*2*^ = 14.374, *df* = 3, *p* = 0.002), but only in the sense that the Stimulating Sounds group contained slightly more pupils as compared to the Neutral Sounds group (51% vs. 32%), and the Neutral Sounds group slightly more individuals who graduated from their studies (40% vs. 26%). There was also a difference in the device used in the study (*χ*^*2*^ = 11.137, *df* = 3, *p* = 0.011), i.e., more individuals used a smartphone in the Stimulating Sounds group than in the Neutral Sounds group (34% vs. 19%). As a consequence, the latter more individuals used a computer (80% vs. 65%). Additionally, the Stimulating Sounds group scored slightly better in the baseline than the Neutral Sounds group. In the case of the Raven's Progressive Matrices test, however, it was only a trend (the mean difference = 5.26, *SE* = 2.93, *t* = 1.795, *df* = 173, *p* = 0.074, *d* = 0.278), while in the case of the Matrix Reasoning Item Bank test, this difference was significant (the mean difference = 7.87, *SE* = 3.61, *t* = 2.181, *df* = 192, *p* = 0.030, *d* = 0.314).

In Study 2 participants were randomly assigned to five groups (called None, Classical, Pure, 5 Hz and 15 Hz after the type of acoustic stimulation). These groups also did not differ when it comes to their basic characteristics, such as age (*F*_4,546_ = 1.460, *p* = 0.213), gender (*χ*^*2*^ = 13.074, *df* = 8, *p* = 0.109), residence (*χ*^*2*^ = 13.047, *df* = 8, *p* = 0.110), status / everyday activity (*χ*^*2*^ = 6.037, *df* = 12, *p* = 0.914) or device used in the study (*χ*^*2*^ = 15.216, *df* = 12, *p* = 0.230). The groups differed with respect to education (*χ*^*2*^ = 25.526, *df* = 12, *p* = 0.013), but only in the sense that the group which carried out the task in silence contained about 15% more individuals who graduated from their studies that the others. By analogy, it contained fewer of those who were still learning. In this study, the differences between the baseline scores were smaller than in Study 1﻿. There were no differences between the groups for the Raven's Progressive Matrices test (*F*_*2,263*_ = 1.450, *p* = 0.218, *η*^*2*^*p* = 0.022). However, in the case of the Matrix Reasoning Item Bank test, there was a trend (*F*_*2,263*_ = 2.320, *p* = 0.057, *η*^*2*^*p* = 0.032), such that the 5 Hz group scored lower than the None group (the mean difference = − 13.02, *SE* = 4.84,* t* = − 2.690, *df* = 278, *Tukey's p* = 0.058, *d* = − 0.520).

Before taking part in the study, the participants were ensured of their anonymity, and all of them provided written informed consent. We also informed partakers about all possible inconveniences linked to participation in the experiment. Moreover, all participants were debriefed. The procedure of this project was approved by The Ethics Committee of the Faculty of Educational Studies at Adam Mickiewicz University, Poznan on December 22, 2021 (Ethical Approval No. WSE-KEdsPB-01a/2021/2022), and was carried out in accordance with the principles of the Helsinki 1964 Declaration and its subsequent amendments.

### Tasks

#### Raven’s Progressive Matrices test

The Raven’s Progressive Matrices test is based on tasks that require inductive reasoning and is used to examine fluid intelligence—a core component of general cognitive ability^43^. We applied its advanced version, used in research with adult participants^58^. It consists of 36 trials. Each has nine geometric patterns organized in a 3 × 3 matrix, arranged according to an unknown set of rules. The bottom right pattern is always missing. The task is to match the correct missing pattern from a set of eight possible matches. Jaeggi et al.^59^ divided these 36 matrices into two equally difficult sets of 18 matrices in order to use the test in research with the baseline measurement, or with a pre- and post-test. Similarly to the procedure that is replicated here, we used the test version with no time restriction^41^. In this version, completing each part takes about half an hour, and a better score is reflected only in the number of attempts that were completed correctly (the time is not compared, because the participant knows that there is no hurry)^60^.

#### Matrix Reasoning Item Bank test

The Matrix Reasoning Item Bank test is a novel, open-access (freely available for non-commercial purposes at https://osf.io/g96f4/) bank of abstract reasoning items designed analogously to Raven’s Progressive Matrices test^61^. Similarly, it consists of a 3 × 3 matrix containing abstract shapes in eight out of nine cells, while one cell on the bottom right-hand side of the matrix is empty. However, unlike in Raven’s test, participants have to identify the missing shape from a set of only four possible alternatives. In addition, the MaRs-IB matrices are coloured and can vary across four dimensions: colour, size, position, and shape. Moreover, there are three sets of counterbalanced tests (test form 1, test form 2, and test form 3) with 80 numbered matrices, differing in each test only in the exact shape used. Therefore, selected test forms can be used as a baseline or pre-test, and as an intervention- or post-test. It is not necessary to use all 80 items. They can be selected according to the particular study's needs (difficulty and task duration). For imitating the rules of Raven’s Advanced Progressive Matrices test, we selected the 18 most difficult matrices from two test forms. In order to do that, we ranked the matrices according to the number of transformations—from the largest to the smallest (the number of transformations, and thus difficulties, for successive matrices between tests is constant, i.e., matrix no. 75 in test form 1 has the same number of transformations as in test forms 2 and 3). Then we selected 18 matrices with the largest number of transformations (from 5 to 8 transformations). These were matrices: 14, 21, 24, 26, 29, 30, 35, 36, 44, 45, 46, 52, 54, 55, 64, 66, 75, 78. Intending to choose the test forms, we used data from a pilot study by the authors of this test shared with the test at https://osf.io/g96f4/. In short, we compared in pairs the average correctness of the answers given to the matrices we selected between the test forms. The smallest differences were noted between test form 2 and test form 3, so we selected 18 matrices (with the numbers indicated above) from test form 2 for the baseline and analogously 18 from test form 3 for the intervention. The Pearson correlation analyses showed that the baseline scores were correlated with the intervention scores regardless of the group type of both our studies (all *p* < 0.00001, see Supplementary Fig. 4 for more details), which indicates the reliability of this test in terms of baseline-intervention comparisons. Since there is no predetermined order of the answers to choose from in a given matrix, we randomized them ourselves. The full set of selected matrices with possible matches dedicated to each of them is available in the Open Science Framework database (https://osf.io/kp48h). Items were presented in the same order for all participants and exactly as in Raven's Matrices, and there was no time limit.

#### Theories of Intelligence scale

The Theories of Intelligence scale evaluates beliefs about the malleability of intelligence^62^. We used the Polish adaptation designed by Kanafa‑Chmielewska^63^. It consists of eight statements that one needs to take a stance on, from strongly agree to strongly disagree, with the use of a seven-point scale. The higher the final score of a participant is, the more they believe in their ability to influence their intelligence.

#### Need for Cognition scale

The Need for Cognition scale measures beliefs regarding cognition, and more precisely, to what extent cognitive activity can be a source of pleasure^64^. We used the Polish adaptation designed by Matusz, Traczyk and Gasiorowska^65^. It consists of 36 statements that one needs to take a stance on, from strongly agree to strongly disagree, with the use of a five-point scale. The higher the final score of a participant is, the more pleasurable cognitive activities are for them, and also the greater their need for cognition is.

### Acoustic stimuli

We used the battery of acoustic stimuli from the study by Beauchene and collaborators^18^. Binaural beats audio files were created using Audacity for Mac (Version 2.4.2). To produce the main experimental condition, i.e., 15 Hz binaural beats (beta band stimulation), two sine tones of 240 and 255 Hz (with the amplitude set to 0.8) were used in the right and left channels, respectively. For the control 5 Hz binaural beats (theta band stimulation), we used 240 and 245 Hz configuration. The pure tone condition was based on two identical sine tones of 240 Hz in both channels. The classical music control condition was the Four Seasons by Vivaldi, in the following order: Spring, Summer, Autumn, Winter. If the participant did not finish the test before all four seasons were played back, the program would start the recording from the beginning. The full set of acoustic stimuli used in our project is available in the Open Science Framework database (https://osf.io/kp48h).

The stimuli were presented to the participants using their stereo headphones, which is common in online acoustic research^66^. However, each participant had to take a test to check that they put the headphones on correctly. It consisted in listening to noise in one of the headphones and indicating whether it was the right or the left one. In case of an error, the headphones had to be readjusted. The volume of the auditory stimuli was set by the participants before starting the test, while listening to the target acoustic stimulus. The instructions indicated that the sound level had to be comfortable.

### Data analyses

All statistical analyses were carried out using *jamovi* for Mac (Version 2.3.18.0)^67–71^. Each test was two-tailed. The adopted level of significance was *α* = 0.05. The analysis of participants’ statements was not based on any quantitative strategy. When reading their statements, we created categories to which each statement was assigned, and we calculated how often each category would be present. As we only aimed at illustrating a trend and being aware that the assignation to a given category may sometimes be erroneous, we did not state exact % values, but rounded-off values. The sample size was calculated using G*Power for Mac (Version 3.1.9.6).

## Supplementary Information

Supplementary Information.

## Data Availability

The anonymised data generated in this study and source data have been deposited in the Open Science Framework database (https://osf.io/kp48h).
