# Supplementary material for: Reverse effect of home-use binaural beats brain stimulation
Source: Sci Rep. 2023 Jul 8;13:11079. doi: 10.1038/s41598-023-38313-4 (PMC10329717; doi:10.1038/s41598-023-38313-4)
Supplement: Supplementary file 1 — Supplementary Information. [file 41598_2023_38313_MOESM1_ESM.pdf]

Supplementary Information

Reverse effect of home-use binaural beats brain stimulation

Michal Klichowski[1,2]* (https://orcid.org/0000-0002-1614-926X)
Andrzej Wicher[1,3] (https://orcid.org/0000-0003-4332-6564)
Agnieszka Kruszwicka[1,2] (https://orcid.org/0000-0001-7834-2316)
Roman Golebiewski[1,3] (https://orcid.org/0000-0001-6712-8154)

[1] Cognitive Neuroscience Center, Adam Mickiewicz University, Poznan, Poland
[2] Learning Laboratory, Faculty of Educational Studies, Adam Mickiewicz University, Poznan, Poland
[3] Department of Acoustics, Faculty of Physics, Adam Mickiewicz University, Poznan, Poland
* email: klich@amu.edu.pl

Table of Contents

Supplementary Table 1 p. 1
Supplementary Figure 1 p. 2
Supplementary Figure 2 p. 2
Supplementary Figure 3 p. 3
Supplementary Table 2 p. 3
Supplementary Table 3 p. 4
Supplementary Table 4 p. 4
Supplementary Table 5 p. 5
Supplementary Figure 4 p. 6

Supplementary Table 1 | Home-use binaural beats brain stimulation affects the score of fluid intelligence test regardless of the test type and group

	Sum of squares	Mean square	F	df	p	η^2p
Time	6133	6132	72.207	1	0.00001***	0.165
Time × Group	63	63	0.737	1	0.391	0.002
Time × Test	272	272	3.198	1	0.075	0.009
Time × Group × Test	198	198	2.330	1	0.128	0.006
Residuals	30999	84		365		

2 × 2 × 2 ANOVA indicating the influence of the binaural beats (15 Hz) intervention on the fluid intelligence test score. Asterisks indicate significant differences with p-values of ***$p < 0.001$ ($n = 369$).

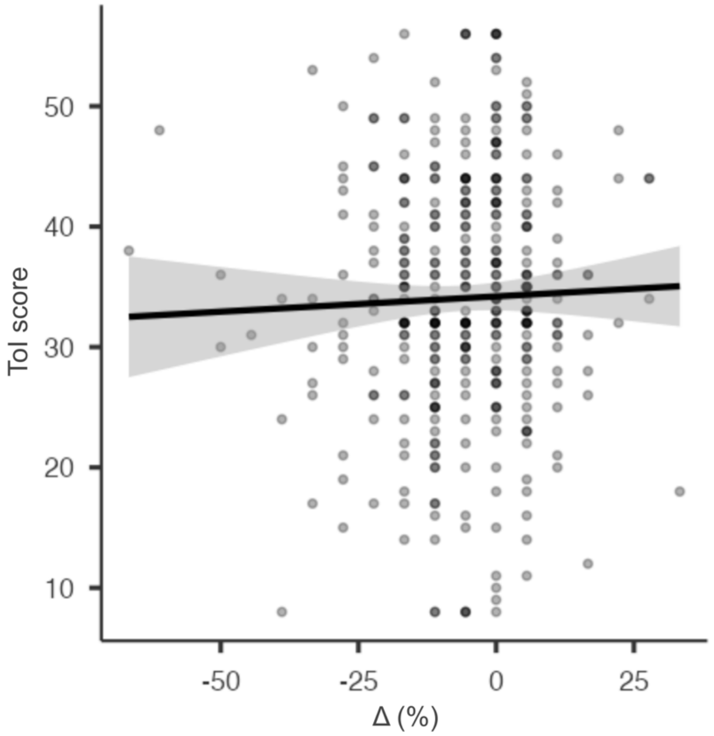

Supplementary Fig. 1 | Correlations between values of the change/delta (Δ) and the values of the Theories of Intelligence scale (ToI). The Pearson correlation analysis showed that the Δ were not correlated with ToI scores (r = 0.032, p = 0.536, n = 369). Source data are provided as a Source Data file deposited in the Open Science Framework database (https://osf.io/kp48h).

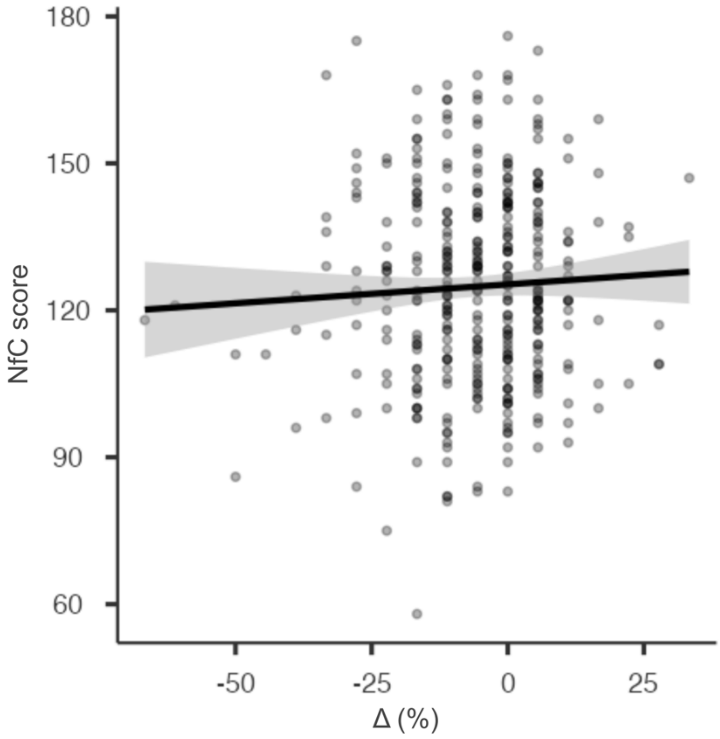

Supplementary Fig. 2 | Correlations between values of the change/delta (Δ) and the values of the Need for Cognition scale (NfC). The Pearson correlation analysis showed that the Δ were not correlated with NfC scores (r = 0.050, p = 0.334, n = 369). Source data are provided as a Source Data file deposited in the Open Science Framework database (https://osf.io/kp48h).

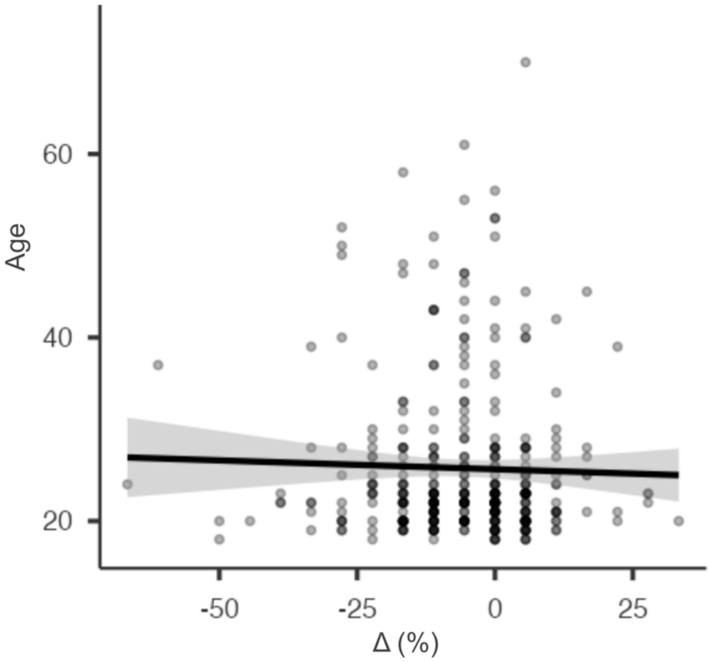

Supplementary Fig. 3 | Correlations between values of the change/delta (Δ) and age. The Pearson correlation analysis showed that the Δ were not correlated with age (r = – 0.028, p = 0.587, n = 369). Source data are provided as a Source Data file deposited in the Open Science Framework database (https://osf.io/kp48h).

Supplementary Table 2 | Home-use binaural beats brain stimulation worsens the scores of both fluid intelligence tests to the same extent regardless of the demographic characteristics

	Sum of squares	Mean square	*F*	*df*	*p*	η^2p
Gender	201	100	0.585	2	0.558	0.003
Residuals	62811	172		366		
Residence	143	72	0.418	2	0.659	0.002
Residuals	62868	171.8		366		
Status	497	166	0.968	3	0.408	0.008
Residuals	62514	171		365		
Education	1082	361	2.120	3	0.097	0.017
Residuals	61930	170		365		
Device	183	61	0.354	3	0.786	0.003
Residuals	62829	172.1		365		

ANOVAs indicating no differences in the influence of the binaural beats (15 Hz) intervention on the fluid intelligence test score by demographic characteristics (n = 369).

Supplementary Table 3 | Home-use of binaural beats brain stimulation is not moderated by the need for cognition and beliefs about intelligence

Scale	Group	Score	*t*	*df*	*p*	*d*
ToI	15 Hz NS (*n* = 157)	33.65 (0.82)	– 0.650	367	0.516	– 0.068
	15 Hz SS (*n* = 212)	34.35 (0.71)				
NfC	15 Hz NS (*n* = 157)	123.34 (1.51)	– 1.238	367	0.217	– 0.130
	15 Hz SS (*n* = 212)	125.95 (1.43)				

Independent samples *t*-test indicating no influence of the need for cognition and beliefs about intelligence on the choice to participate in a study on brain stimulation. *ToI* Theories of Intelligence scale, *NfC* Need for Cognition scale, *NS* neutral sounds, *SS* stimulating sounds. Standard errors are in parentheses.

Supplementary Table 4 | Justifications for the study choice

Study type	Justification categories	Justification examples
Neutral Sounds	Curiosity about the study course	“it seemed more interesting to me and attracted my attention more”<br>“I was curious to know what the standard study looks like”
	Willingness to carry out an easier task	“I assumed this study would be easier to complete”
	Afraid of brain stimulation	“brain stimulation made me anxious”<br>“brain stimulation sounded discouraging”
	Difficult to classify	“I chose the standard study because I assumed that it would suit me better”<br>“I like standards”
Stimulating Sounds	Cognitive curiosity about brain stimulation	“I am fascinated by how the human brain works and I wanted to see if there are actual methods of brain stimulation and whether it would have any effect on me”<br>“I wanted to see if brain stimulation would really work”<br>“brain stimulation seemed a more attractive and unique version of the study”<br>“I was curious to check what brain stimulation would consist in”<br>“brain stimulation seemed like a definitely more interesting solution”<br>“brain stimulation sounded more interesting”<br>“I found the name of the study itself very interesting”<br>“I chose brain stimulation because it seemed very interesting”<br>“the notion of brain stimulation seemed interesting, which is why I decided to choose it”
	Need for cognitive challenges	“I don’t like standard/simple things, I prefer toiling a little”<br>“I like challenges and more difficult tasks”<br>“I prefer a more complicated and interesting option”<br>“I wanted to try a study that would require thinking”<br>“a more engaging task sounded more interesting”

No answer	“.”
	“…”
Difficult to classify	“I decided that it was appropriate”
	“judging by the description it seemed to me that the standard test would include too many sounds that I expected to be irritating for me, which I wanted to avoid”
	“I chose this study because I didn’t know what I could expect from the other one”
	“I decided this way”
	“I chose this option because it was more thoroughly described than the other”
	“it seemed more accessible to me”
	“I thought that this study would suit me better”

Feedback added to justification that the stimulation interfered with the task: “after hearing the sound I was unable to focus on the task”, “the sound that I heard disturbed me in carrying out further tasks”.

Supplementary Table 5 | Home-use binaural beats brain stimulations affects the scores of fluid intelligence tests in contrast to other acoustic stimulations

	**Sum of squares**	**Mean square**	***F***	***df***	***p***	$\eta^2 p$
Time	1959	1959	21.959	1	0.00001***	0.039
Time × Group	2246	561	6.295	4	0.00006***	0.044
Time × Test	140	140	1.564	1	0.212	0.003
Time × Group × Test	342	85	0.957	4	0.430	0.007
Residuals	48258	89		541		

2 × 5 × 2 ANOVA indicating the main differences in the influence of the type of intervention on the fluid intelligence test score by group and test type. Asterisks indicate significant differences with *p*-values of ***$p < 0.001$ ($n = 551$).

**Post-hoc comparisons**		**Mean difference (*SE*)**	***t***	***df***	**Tukey's *p***
Baseline	None ($n = 101$)	0.01 (1.33)	0.009	541	1.000
	Classical ($n = 95$)	0.90 (1.37)	0.657	541	1.000
	Pure ($n = 135$)	0.22 (1.15)	0.189	541	1.000
	5 Hz ($n = 114$)	6.23 (1.25)	04.976	541	0.00004***
	15 Hz ($n = 106$)	6.09 (1.30)	04.674	541	0.00016***

Estimated-marginal-means-based post-hoc comparisons indicating the differences in the influence of the type of intervention on the score of fluid intelligence tests. Standard errors are in parentheses. Asterisks indicate significant differences with *p*-values of ***$p < 0.001$.

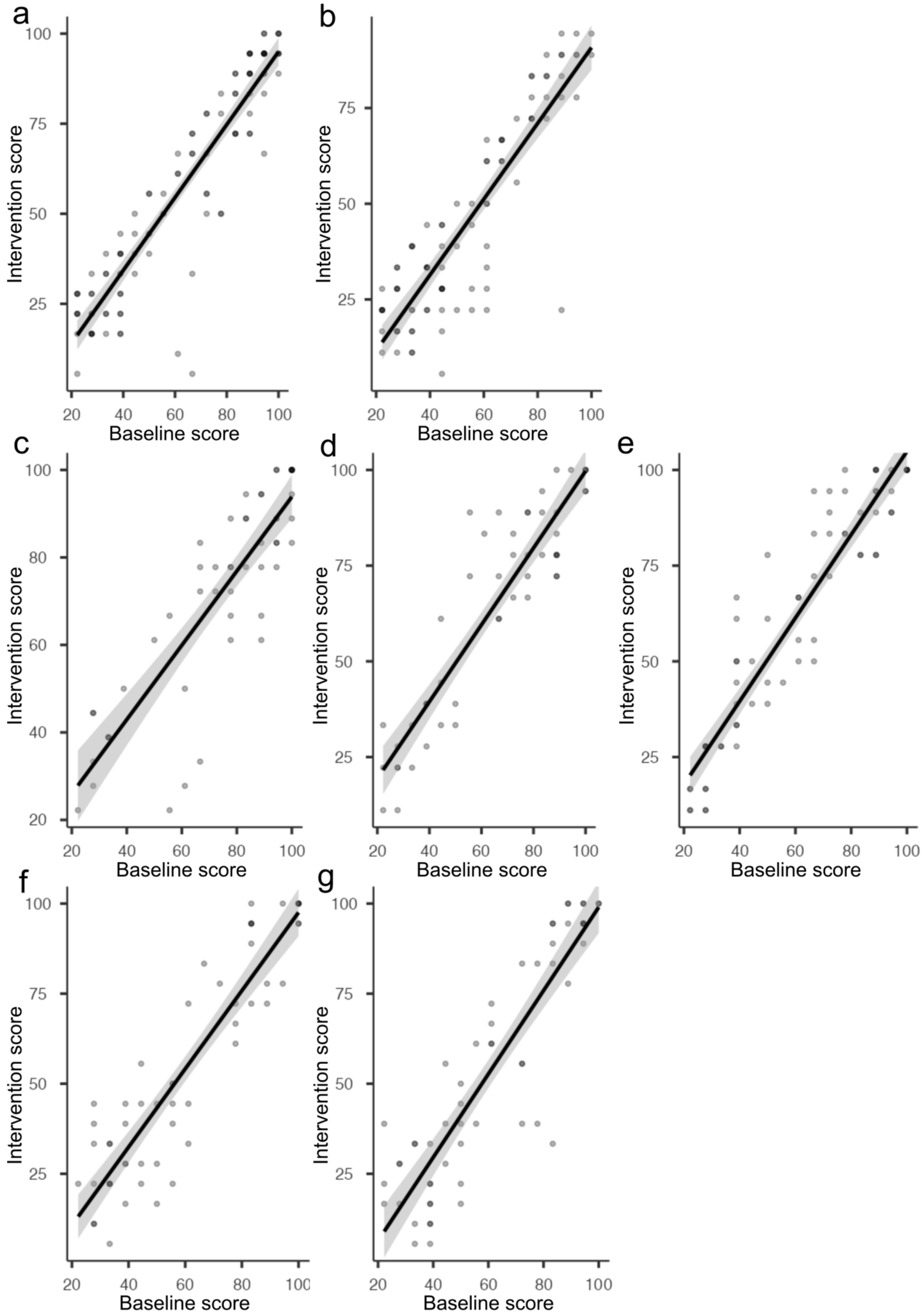

Supplementary Fig. 4 | Correlations between values of the baseline part of Matrix Reasoning Item Bank test and the values of its intervention part. a The Pearson correlation analysis showed that the baseline scores were correlated with the intervention scores in the Neutral Sounds group of Study 1 (r =

0.879, $p < 0.00001$, $n = 89$). **b** The Pearson correlation analysis showed that the baseline scores were correlated with the intervention scores in the Stimulating Sounds group of Study 1 ($r = 0.920$, $p < 0.00001$, $n = 105$). **c** The Pearson correlation analysis showed that the baseline scores were correlated with the intervention scores in the None group of Study 2 ($r = 0.864$, $p < 0.00001$, $n = 52$). **d** The Pearson correlation analysis showed that the baseline scores were correlated with the intervention scores in the Classical group of Study 2 ($r = 0.916$, $p < 0.00001$, $n = 50$). **e** The Pearson correlation analysis showed that the baseline scores were correlated with the intervention scores in the Pure group of Study 2 ($r = 0.932$, $p < 0.00001$, $n = 68$). **f** The Pearson correlation analysis showed that the baseline scores were correlated with the intervention scores in the 5 Hz group of Study 2 ($r = 0.909$, $p < 0.00001$, $n = 55$). **g** The Pearson correlation analysis showed that the baseline scores were correlated with the intervention scores in the 15 Hz group of Study 2 ($r = 0.893$, $p < 0.00001$, $n = 58$). Source data are provided as a Source Data file deposited in the Open Science Framework database (https://osf.io/kp48h).
